# DHA-dominant Omega-3 compositions enhance PSD-95 organization and glutamatergic protein expression

**DOI:** 10.3389/fnsyn.2026.1947075

**Published:** 2026-09-22

**Authors:** Gerardo Medina, Stephen D. Baird, Lilia A. Rodriguez-Alcocer, Alex E. MacKenzie

**Affiliations:** 1 Children’s Hospital of Eastern Ontario Research Institute, Ottawa, ON, Canada; 2 Department of Cellular and Molecular Medicine, Faculty of Medicine, University of Ottawa, Ottawa, ON, Canada; 3 Eric Poulin Center for Neuromuscular Disease, Faculty of Medicine, University of Ottawa, Ottawa, ON, Canada

**Keywords:** docosahexaenoic acid, eicosapentaenoic acid, postsynaptic density, postsynaptic density protein-95, synaptic plasticity

## Abstract

**Introduction:**

Docosahexaenoic acid (DHA), the predominant omega-3 (ω-3) fatty acid in the brain, which contributes to synaptic membrane organization and plasticity, is commonly recommended for brain health. However, ω-3 formulations commonly combine DHA with eicosapentaenoic acid (EPA) in varying proportions, and the impact of the DHA ratio on postsynaptic scaffold regulation remains unclear.

**Methods:**

In this study, differentiated human SH-SY5Y neurons were cultured in the presence of DHA, EPA, or defined DHA ratios (2:1, 1:1, 1:2) at equivalent total concentrations. PSD-95 expression, puncta density, colocalization with the presynaptic marker synaptophysin (SYN), and glutamatergic signaling proteins were quantified by immunofluorescence and Western blot analysis. Metabolic activity was assessed using a colorimetric MTT assay.

**Results:**

DHA produced the largest increase in PSD-95 protein abundance by immunoblot and the strongest effects on PSD-95 puncta density and SYN colocalization, accompanied by coordinated upregulation of N-methyl-D-aspartate receptor (NMDAR), α-amino-3-hydroxy-5-methyl-4-isoxazolepropionic acid receptor (AMPAR), and calcium/calmodulin-dependent protein kinase II (CaMKII). DHA-enriched compositions preserved this profile, whereas balanced or EPA-dominant compositions showed attenuated responses relative to DHA-dominant conditions. EPA alone failed to enhance postsynaptic scaffold abundance and reduced NMDAR levels relative to control. These effects occurred without a detectable reduction in cellular metabolic activity.

**Discussion:**

Collectively, these findings demonstrate that the composition of ω-3 fatty acid has a significant bearing on postsynaptic scaffold organization and glutamatergic signaling in differentiated neurons, with DHA serving as the chief mediator of observed effects. This composition-dependent framework provides an experimental basis for considering DHA ratio when evaluating omega-3 effects on postsynaptic protein organization.

## 1 Introduction

Docosahexaenoic acid (DHA; 22:6n-3) is the major long-chain omega-3 (ω-3) polyunsaturated fatty acid (PUFA) in the central nervous system (CNS) and is highly enriched in neuronal membranes, particularly in gray matter (Brenna and Carlson, 2014; Carver et al., 2001; Singh, 2005; Söderberg et al., 1991). DHA is predominantly esterified into synaptic phospholipids, most notably phosphatidylserine (PS) and phosphatidylethanolamine (PE), where it contributes to membrane curvature, acyl chain disorder, and the lateral organization of membrane microdomains (Brenna and Diau, 2007; Kim et al., 2022; Suzuki et al., 1997; Westra et al., 2021). These properties enable DHA to regulate synaptic membrane structure, influencing receptor localization, protein-lipid interactions, and excitatory synapse organization (Sherratt and Mason, 2018; Westra et al., 2021). During development, DHA supports neuronal differentiation, neurite extension, and synaptogenesis (Brenna and Carlson, 2014; Calder, 2016; Cao et al., 2009; Innis, 2008). In mature neurons, it modulates membrane fluidity and lipid domain composition, facilitating clustering and stabilization of glutamatergic receptors, including N-methyl-D-aspartate receptors (NMDARs) and α-amino-3-hydroxy-5-methyl-4-isoxazolepropionic acid receptors (AMPARs) (Cao et al., 2009; Sherratt and Mason, 2018; Sherratt et al., 2021; Westra et al., 2021).

In contrast, another major long-chain ω-3 fatty acid, eicosapentaenoic acid (EPA; 20:5n-3), exhibits distinct metabolic handling and membrane distribution within the brain. Under physiological conditions, brain EPA levels remain comparatively low and are characterized by rapid turnover (Chen et al., 2009; Chen et al., 2013; Kaur et al., 2013), consistent with a limited contribution to membrane lipid composition. EPA has also been reported to modulate phosphoinositide 3-kinase (PI3K) signaling in neuronal models (Kawashima et al., 2010), suggesting a more dynamic and functionally distinct role compared with the structural enrichment observed for DHA.

Clinically, ω-3 fatty acid formulations are widely used across a range of conditions, including cardiovascular disease, inflammation, and mood-related disorders, typically as combined DHA and EPA formulations (Calder, 2015; Mozaffarian and Wu, 2011; Sublette et al., 2011). The DHA:EPA ratio in such formulations varies substantially; in addition, the relative contribution of DHA and EPA to synaptic or postsynaptic processes remains unclear. When present together, DHA and EPA may undergo distinct metabolic handling and occupy different membrane environments, potentially modifying the resulting membrane organization (Dyall, 2015; Sherratt and Mason, 2018; Sherratt et al., 2021). These properties indicate that ω-3 fatty acids are not functionally interchangeable at the synapse, and that the proportions of DHA and EPA in a given ω-3 formulation may materially influence postsynaptic scaffold regulation and synaptic organization.

A key postsynaptic scaffold protein is postsynaptic density protein-95 (PSD-95), which anchors NMDARs and AMPARs and organizes downstream signaling pathways. By linking receptor localization with central signaling proteins such as calcium/calmodulin-dependent protein kinase II (CaMKII), PSD-95 integrates synaptic structure with activity-dependent plasticity (Nicoll and Schulman, 2023; Petralia et al., 2005; Swulius et al., 2010; Yasuda et al., 2022). PSD-95 therefore represents a functionally relevant point at which lipid composition can influence postsynaptic scaffold regulation and glutamatergic signaling (Westra et al., 2021).

Consistent with its role in synaptic plasticity, PSD-95 disruption is implicated in various neurodevelopmental and neurodegenerative disorders, including DLG4-related synaptopathy, caused by pathogenic variants in the gene encoding PSD-95 (Rodríguez-Palmero et al., 2021). Alterations in PSD-95 expression and scaffold organization are also reported in autism spectrum disorder, schizophrenia, Alzheimer’s disease, and Fragile X syndrome (Funk et al., 2017; Kindler and Kreienkamp, 2012; Levy et al., 2022; Medina and MacKenzie, 2024; Yuki et al., 2014), in which impaired glutamatergic receptor organization and synaptic plasticity represent shared features. Together, these findings support the investigation of PSD-95 modulation as a potential therapeutic avenue in disorders characterized by postsynaptic scaffold dysfunction.

Notably, DHA has been shown to modulate postsynaptic architecture, including PSD-95 expression and localization. DHA supplementation increases PSD-95 expression and promotes synaptic protein organization in neuronal systems (Calon et al., 2004; Cao et al., 2009; Sidhu et al., 2011), whereas ω-3 deficiency is associated with reduced PSD-95 levels and disrupted synaptic organization (Aryal et al., 2019; Madore et al., 2020; Sidhu et al., 2016). However, these observations have largely been derived from DHA-centric or deficiency paradigms, and it remains unclear whether such effects are preserved, attenuated, or modified when DHA is present in combination with EPA. In particular, the extent to which defined DHA:EPA molar ratios differentially regulate PSD-95-dependent postsynaptic organization remains unclear.

To address this gap, we used differentiated SH-SY5Y cells as a human neuronal-like model that permits controlled analysis of PSD-95 organization and associated glutamatergic proteins (Shipley et al., 2016; Kovalevich et al., 2021). We examined the effects of defined DHA:EPA molar ratios (2:1, 1:1, and 1:2) on PSD-95 expression, PSD-95-SYN colocalization, and associated glutamatergic signaling proteins. The 2:1, 1:1, and 1:2 ratios were selected to provide a symmetric composition series spanning DHA-dominant, equimolar, and EPA-dominant ratios between the DHA-only and EPA-only treatment endpoints. We tested whether DHA:EPA ratio regulates PSD-95-dependent postsynaptic organization.

## 2 Materials and methods

### 2.1 Cell culture

Human SH-SY5Y neuroblastoma cells were maintained in a 1:1 mixture of Eagle’s Minimum Essential Medium (EMEM; ATCC) and Ham’s F12 (Thermo Fisher Scientific), supplemented with 10% fetal bovine serum and 1% penicillin–streptomycin. Cultures were kept at 37 °C in a humidified incubator with 5% CO_2_. Media were replaced every 2–3 days, and cells were passaged at approximately 80% confluence.

### 2.2 Differentiation, treatment and immunofluorescence

For neuronal differentiation, SH-SY5Y cells were seeded at a density of 9 × 10^4^ cells/cm^2^ onto Matrigel-coated 96-well plates and treated with 10 μM all-trans retinoic acid (Sigma-Aldrich) for 7 days. Cells were subsequently maintained for an additional 7 days in BrainPhys™ Neuronal Medium (STEMCELL Technologies) supplemented with SM1 to promote neuronal maturation. This 14-day differentiation period is referred to as days *in vitro* (DIV14).

After 14 days of differentiation, cells were treated for 24 h with DHA (50 μM), EPA (50 μM), or DHA:EPA mixtures at molar ratios of 2:1 (33.3 μM DHA + 16.7 μM EPA), 1:1 (25 μM DHA + 25 μM EPA), or 1:2 (16.7 μM DHA + 33.3 μM EPA) (all fatty acids from Cayman Chemical), maintaining a total fatty acid concentration of 50 μM across treatment conditions. The 50 μM concentration was selected as an experimental exposure with precedent in human neuronal culture (Zhao et al., 2019) and was not intended to reproduce a specific circulating, brain, or dietary concentration. Vehicle control cells received an equivalent concentration of EtOH (final concentration ≤0.04%), and untreated cells were included as an additional control group. Fatty acids were dissolved in EtOH and diluted into differentiation medium immediately before use. The final EtOH concentration was kept constant across treatment groups. Together with the DHA-only and EPA-only conditions, these ratios provided a symmetric five-condition compositional series spanning the DHA-to-EPA range while maintaining a constant total fatty acid concentration.

For immunofluorescence analyses, following treatment, cells were fixed with 4% formaldehyde for 30 min and washed three times with phosphate-buffered saline (PBS; 5 min per wash). Cells were permeabilized with 0.1% Triton X-100 (Sigma-Aldrich, Cat. No. X100) and blocked with Dako Protein Block Serum-Free Ready-To-Use (Thermo Fisher Scientific; Cat. No. X0909). Cultures were incubated overnight at 4 °C with primary antibodies diluted in blocking solution. For PSD-95/MAP2 staining, cells were incubated with anti-PSD-95 (4 μg/mL; Abcam, Cambridge, UK; Cat. No. ab18258) and anti-MAP2 (1:450; Invitrogen, Cat. No. 13–1,500), followed by anti-rabbit Alexa Fluor 488 (1:1000; Abcam, Cat. No. ab300671) and goat anti-mouse IgG H&L Alexa Fluor 594 (1:1000; Abcam, Cat. No. ab150116) secondary antibodies for 1 h at room temperature. For PSD-95/SYN colocalization analyses, PSD-95 was detected using FluoTag®-X2 anti-PSD95 conjugated with Alexa Fluor 647 (1:50; NanoTag Biotechnologies; Cat. No. N3702), together with anti-synaptophysin (1:500; Thermo Fisher Scientific; Cat. No. PA5-27286), followed by anti-rabbit Alexa Fluor 488 (1:1000; Abcam, Cat. No. ab300671) for 1 h at room temperature. Nuclei were counterstained with Hoechst (2.5 μg/mL; Invitrogen, Cat. No. H3570).

### 2.3 MTT metabolic activity assay

Metabolic activity following fatty acid treatment was assessed using a colorimetric MTT assay (Sigma-Aldrich). Differentiated SH-SY5Y cells were treated for 24 h as described above. MTT reagent (0.5 mg/mL final concentration) was added to each well and incubated for 90 min at 37 °C. Formazan crystals were solubilized in DMSO, and absorbance was measured at 570 nm using a microplate reader. MTT absorbance values were expressed relative to vehicle-treated controls. Each condition was tested in three independent biological replicates.

### 2.4 Image acquisition and quantitative analysis

Immunofluorescence images were acquired using an Opera Phenix High Content Screening System (PerkinElmer). For each well, 16–20 non-overlapping fields were captured using identical exposure settings across treatment groups within each biological replicate. No post-acquisition contrast adjustments were applied before analysis.

Image analysis was performed using Columbus software (PerkinElmer) following a standardized segmentation pipeline. Nuclei were identified from Hoechst staining using fixed-intensity thresholds. Cytoplasmic regions were defined by expansion from the nuclear mask into MAP2- or SYN-positive areas. For the original whole-cell fluorescence analysis, quantitative measurements were restricted to the perinuclear soma compartment to minimize variability arising from neuritic morphology. To assess PSD-95 within neuronal processes, MAP2-positive neurites were additionally segmented using the Columbus neurite-analysis module. PSD-95 fluorescence intensity was quantified within the resulting neurite segments, and PSD-95 puncta detected within the MAP2-positive neurite compartment were normalized to total neurite length and expressed as puncta per 100 μm of neurite.

PSD-95 and SYN mean intensities were calculated on a per-cell basis within the defined cytoplasmic region. For puncta analysis, PSD-95-positive puncta were detected using fixed intensity and size thresholds established during initial pipeline optimization and subsequently applied identically across all wells and biological replicates. Puncta number and mean puncta intensity were quantified per cell and averaged per well.

PSD-95-SYN colocalization was quantified using a pixel-wise overlap approach. Briefly, the PSD-95 and SYN fluorescence channels were multiplied to generate an overlap image, and the mean overlap intensity was measured within the cytoplasmic region of each cell. Overlap values were normalized to the individual channel intensities to approximate Manders’ overlap coefficients and provide a relative measure of spatial colocalization.

### 2.5 Western blot analysis

SH-SY5Y cells were seeded at 5 × 10^5^ cells per well in 6-well plates and allowed to adhere for 24 h. Differentiated cultures were then treated for 24 h with vehicle control, DHA (50 μM), EPA (50 μM), or DHA:EPA mixtures at molar ratios of 2:1, 1:1, and 1:2, as described above. Each treatment condition was evaluated in three independent biological replicates derived from separate cell passages differentiated and treated on different days. Independent protein lysates were prepared for each replicate and processed on separate gels and membranes. The immunoblots shown in the figures are representative of the three independent experiments.

Following treatment, cells were lysed on ice in RIPA-based lysis buffer supplemented with Halt™ Protease and Phosphatase Inhibitor Cocktail (Thermo Scientific, Cat. No. 1861281) for 20–30 min. Lysates were collected by scraping, transferred to microcentrifuge tubes, briefly sonicated, and centrifuged at 13,000 × g for 30 min at 4 °C. Supernatants were collected, and total protein concentration was determined using a Bradford-based protein assay with BSA standards.

Equal amounts of protein (10 μg per lane) were mixed with Laemmli sample buffer (BioRad) containing 1% β-mercaptoethanol (Sigma-Aldrich) and denatured at 95 °C for 5 min. Proteins were separated on 4–20% Mini-PROTEAN TGX Stain-Free gels (Bio-Rad, Cat. No. 456–8,096) at 110 V and transferred to low-fluorescence PVDF membranes using the Trans-Blot Turbo system (Bio-Rad) according to the manufacturer’s instructions. Stain-free gels were activated under UV illumination to enable total protein visualization prior to transfer.

Membranes were blocked in 5% non-fat milk in TBS-T (0.1% Tween-20) for 30–60 min at room temperature and incubated with primary antibodies for 1 h at room temperature: anti-PSD-95 (rabbit; Abcam, Cat. No. ab18258, 3 μg/mL), anti-Glutamate Receptor 1 (AMPA subtype) antibody (rabbit; Abcam, Cat. No. ab109450, 1:2000), anti-NMDAR2B antibody (mouse; Abcam, Cat. No. ab93610, 1:250) and Anti-CaMKII antibody (rabbit; Abcam, Cat. No. ab134041, 1:1000). After washing in TBS-T, membranes were incubated for 1 h at room temperature with the appropriate HRP-conjugated secondary antibodies: goat anti-rabbit IgG (H + L)-HRP (Thermo Fisher Scientific, Cat. No. 31460, 1:5,000) and goat anti-mouse IgG (H + L)-HRP (Thermo Fisher Scientific, Cat. No. 31430, 1:5,000).

Chemiluminescent signals were detected using a ChemiDoc imaging system (Bio-Rad), and band intensities were quantified by densitometry using ImageLab software (Bio-Rad). Target protein levels were normalized to the total protein signal from the corresponding stain-free membrane lane (Supplementary Figure 1). Total-protein normalization was intentionally selected because it provides a lane-wide measure of protein loading that accounts for loading variability across lanes without relying on a single housekeeping protein, and it was applied consistently across all treatment groups (Gilda and Gomes, 2013; Maloy et al., 2022).

### 2.6 Statistical analysis

Statistical analyses were performed using GraphPad Prism version 8.4.2 (GraphPad Software, San Diego, CA). For all datasets, including MTT, immunofluorescence, puncta quantification, apposition analysis, and Western blot densitometry, group differences were evaluated using one-way analysis of variance (ANOVA) followed by Tukey’s multiple comparisons *post hoc* test. Normality of data distributions was assessed using the Shapiro–Wilk test in GraphPad Prism before parametric testing.

For Western blot analyses, each independent membrane contained the complete treatment set together with its matched vehicle control. Because absolute chemiluminescent signal intensities cannot be directly compared across membranes processed on separate days, target protein signals were first normalized to the corresponding lane total-protein signal and then expressed relative to the matched vehicle control within each biological replicate. The resulting within-experiment fold changes were pooled across the three independent biological replicates for statistical comparison. Accordingly, the Western blot graphs represent relative treatment responses across independent experiments rather than absolute inter-membrane signal variance.

Data are presented as mean ± standard deviation (SD). Statistical significance was defined as *p* < 0.05.

## 3 Results

### 3.1 DHA preferentially modulates PSD-95 abundance, puncta density, and SYN colocalization

Differentiated SH-SY5Y neurons (DIV14) were exposed for 24 h to DHA (50 μM), EPA (50 μM), or defined DHA:EPA ratios (2:1, 1:1, 1:2; total 50 μM), with vehicle-treated cells used as controls. No significant differences in MTT-derived metabolic activity were observed across treatment groups following 24 h exposure (Supplementary Figure 2).

Immunofluorescence analysis showed that DHA produced the strongest PSD-95 signal and EPA the weakest, while the mixed DHA:EPA ratios displayed intermediate responses relative to DHA treatment (Figures 1A,B). Western blot analysis demonstrated a similar pattern, with DHA producing the largest increase in PSD-95 protein levels relative to control, while EPA treatment had minimal effect (Figures 1C,D). Among the DHA:EPA ratios, the 2:1 ratio most closely resembled DHA alone, whereas the 1:1 and 1:2 compositions showed progressively lower PSD-95 expression.

**Figure 1:**
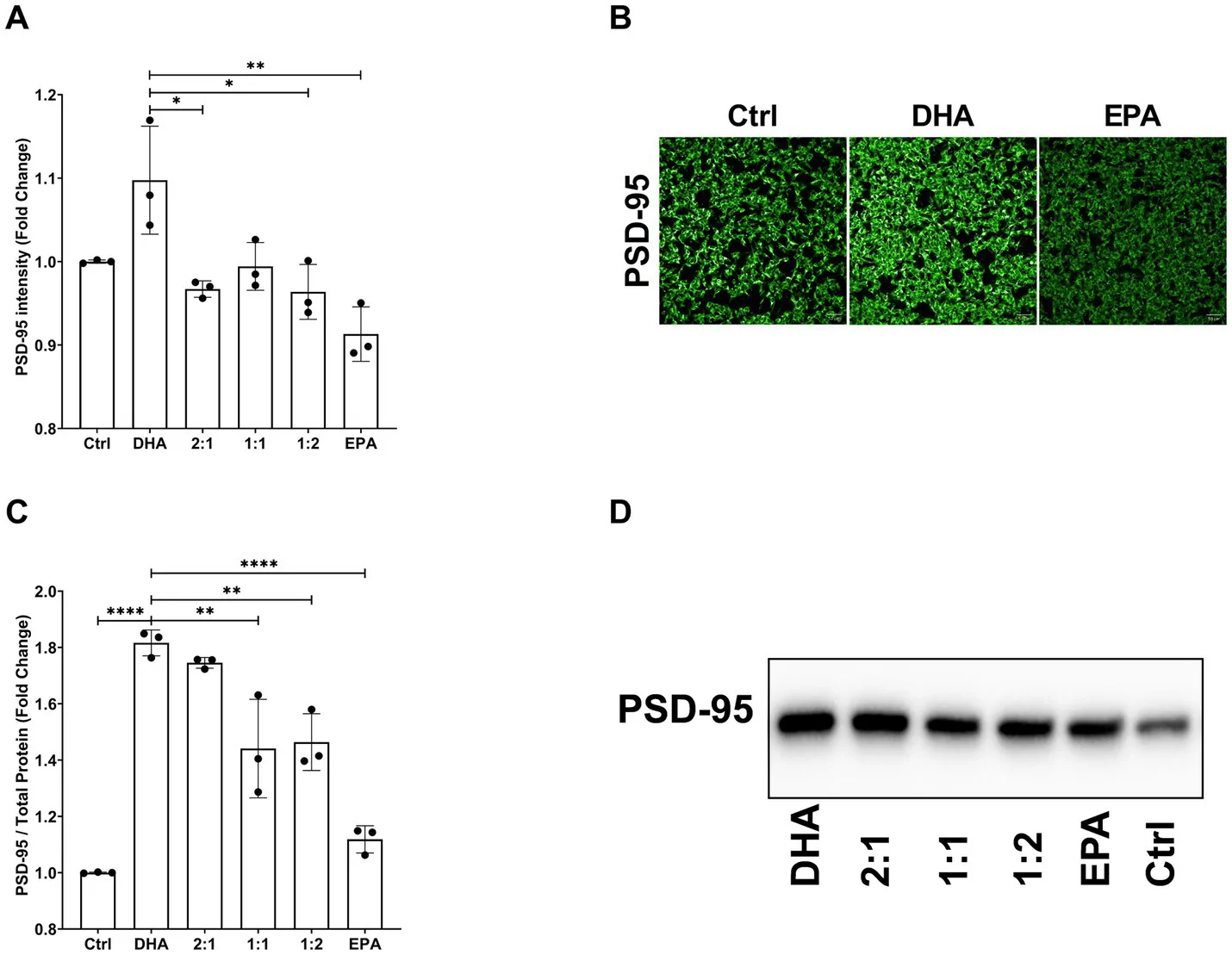
DHA preferentially increases PSD-95 expression in differentiated SH-SY5Y neurons. **(A)** PSD-95 fluorescence intensity in differentiated SH-SY5Y neurons following 24 h treatment with vehicle control (Ctrl), DHA (50 μM), EPA (50 μM), or DHA:EPA ratios at molar ratios of 2:1, 1:1, and 1:2 (total fatty acid concentration 50 μM). DHA increased PSD-95 fluorescence intensity relative to EPA and the 2:1 and 1:2 DHA:EPA ratios. **(B)** Representative immunofluorescence images of PSD-95 (green) in control-, DHA-, and EPA-treated neurons after 24 h exposure. Images were acquired using identical exposure settings across conditions. Scale bar = 50 μm. **(C)** PSD-95 protein levels normalized to total protein following 24 h treatment. DHA increased total PSD-95 protein expression relative to control and EPA, whereas the 1:1 and 1:2 DHA:EPA ratios showed lower PSD-95 levels than DHA alone. **(D)** A representative immunoblot is shown (lanes: DHA, 2:1, 1:1, 1:2, EPA, Ctrl). Data are presented as mean ± SD. Individual points represent independent biological replicates (*n* = 3). Statistical analysis was performed using one-way ANOVA followed by Tukey’s multiple comparisons test. ^*^p < 0.05, ^**^p < 0.01, ^****^p < 0.0001. Only significant pairwise comparisons are shown.

PSD-95 puncta density and colocalization with the presynaptic marker SYN were next examined in differentiated SH-SY5Y neurons following 24 h lipid treatment, with representative immunofluorescence images shown in Figure 2A. Consistent with the immunoblot and immunofluorescence findings above, DHA produced the strongest increase in PSD-95 puncta density relative to control (adjusted *p* = 0.0005), while EPA treatment showed little effect relative to control (Figure 2B). DHA:EPA ratios produced intermediate mean responses, with the 2:1 ratio numerically most closely resembling DHA alone. A similar composition-dependent pattern was observed for PSD-95 colocalization with SYN-positive regions (Figures 2C,D). DHA treatment resulted in the largest increase in total PSD-95 colocalization with SYN relative to control (adjusted *p* < 0.0001), whereas EPA showed minimal change relative to control (Figure 2C). Analysis of the proportion of PSD-95 colocalized with SYN further demonstrated that DHA increased PSD-95 colocalization relative to control (adjusted *p* = 0.0214), whereas EPA reduced it (adjusted *p* = 0.0079) (Figure 2D). Across both colocalization measurements, DHA-enriched compositions retained partial effects, while balanced and EPA-dominant formulations showed attenuated responses.

**Figure 2:**
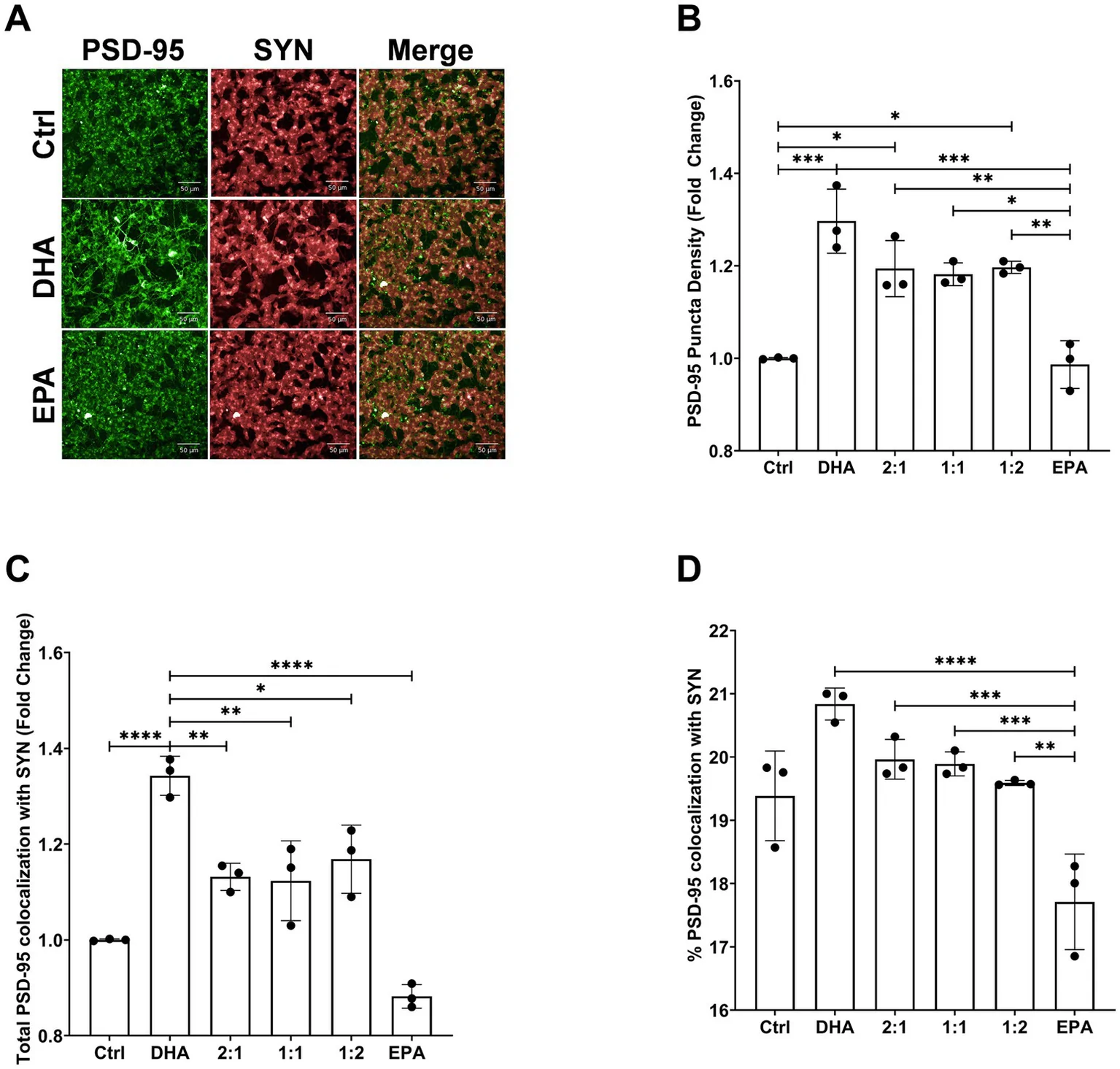
DHA and defined DHA:EPA ratios modulate PSD-95 puncta density and synaptic apposition with Synaptophysin. **(A)** Representative immunofluorescence images of PSD-95 (green), Synaptophysin (SYN; red), and merged channels in differentiated SH-SY5Y neurons treated for 24 h with vehicle control (Ctrl), DHA (50 μM), or EPA (50 μM). Images were acquired using identical exposure and analysis settings across conditions. Scale bar = 50 μm. **(B)** PSD-95 puncta density following 24 h treatment with DHA (50 μM), EPA (50 μM), or DHA:EPA ratios at molar ratios of 2:1, 1:1, and 1:2 (total fatty acid concentration 50 μM). DHA and DHA-containing compositions increased PSD-95 puncta density relative to EPA-treated cells. **(C)** Total PSD-95 signal colocalized with SYN-positive regions following lipid treatment. DHA increased PSD-95-SYN colocalization relative to control and EPA-treated cells. **(D)** Percentage of PSD-95 signal colocalized with SYN-positive regions following 24 h treatment. DHA-containing conditions showed increased PSD-95 localization to SYN-positive sites relative to EPA-treated cells. Data are presented as mean ± SD. Individual points represent independent biological replicates (*n* = 3). Statistical analysis was performed using one-way ANOVA followed by Tukey’s multiple comparisons test. ^*^p < 0.05, ^**^p < 0.01, ^***^p < 0.001, ^****^p < 0.0001. Only significant pairwise comparisons are shown.

To determine whether the PSD-95 response extended into neuronal processes, PSD-95 intensity and puncta density were quantified within MAP2-positive neurites. PSD-95 intensity within MAP2-positive neurites was significantly increased relative to control across all lipid-treated conditions, with the largest increase observed following DHA treatment (adjusted *p* < 0.0001; Supplementary Figures 3A,B). More importantly, PSD-95 puncta density normalized to neurite length was significantly increased by DHA (adjusted *p* < 0.0001), 2:1 DHA:EPA (adjusted *p* = 0.0002), and 1:1 DHA:EPA (adjusted *p* = 0.0076) relative to control, whereas the 1:2 and EPA conditions did not differ significantly from control (Supplementary Figure 3C). These findings indicate that the DHA-associated increase in PSD-95 puncta extends to MAP2-positive neuronal processes and is not restricted to the somatic compartment.

Taken together, these findings indicate that both PSD-95 clustering and PSD-95-SYN colocalization vary with DHA:EPA ratio, with DHA accounting for the majority of the observed ω-3-dependent effects on postsynaptic organization.

### 3.2 Omega-3 modulation of glutamate receptor and CaMKII abundance

NMDAR, AMPAR, and CaMKII, proteins relevant to glutamatergic signaling and synaptic plasticity, showed distinct responses across DHA:EPA ratios (Figure 3A). DHA produced the strongest increase in NMDAR expression relative to control (adjusted *p* < 0.0001), while EPA significantly reduced NMDAR levels relative to control (adjusted *p* = 0.0056) (Figure 3B). Among the DHA:EPA ratios, the 2:1 condition most closely resembled DHA treatment, whereas the 1:1 condition produced a smaller increase and the 1:2 condition did not differ significantly from control, indicating progressively reduced NMDAR expression with increasing EPA proportion.

**Figure 3:**
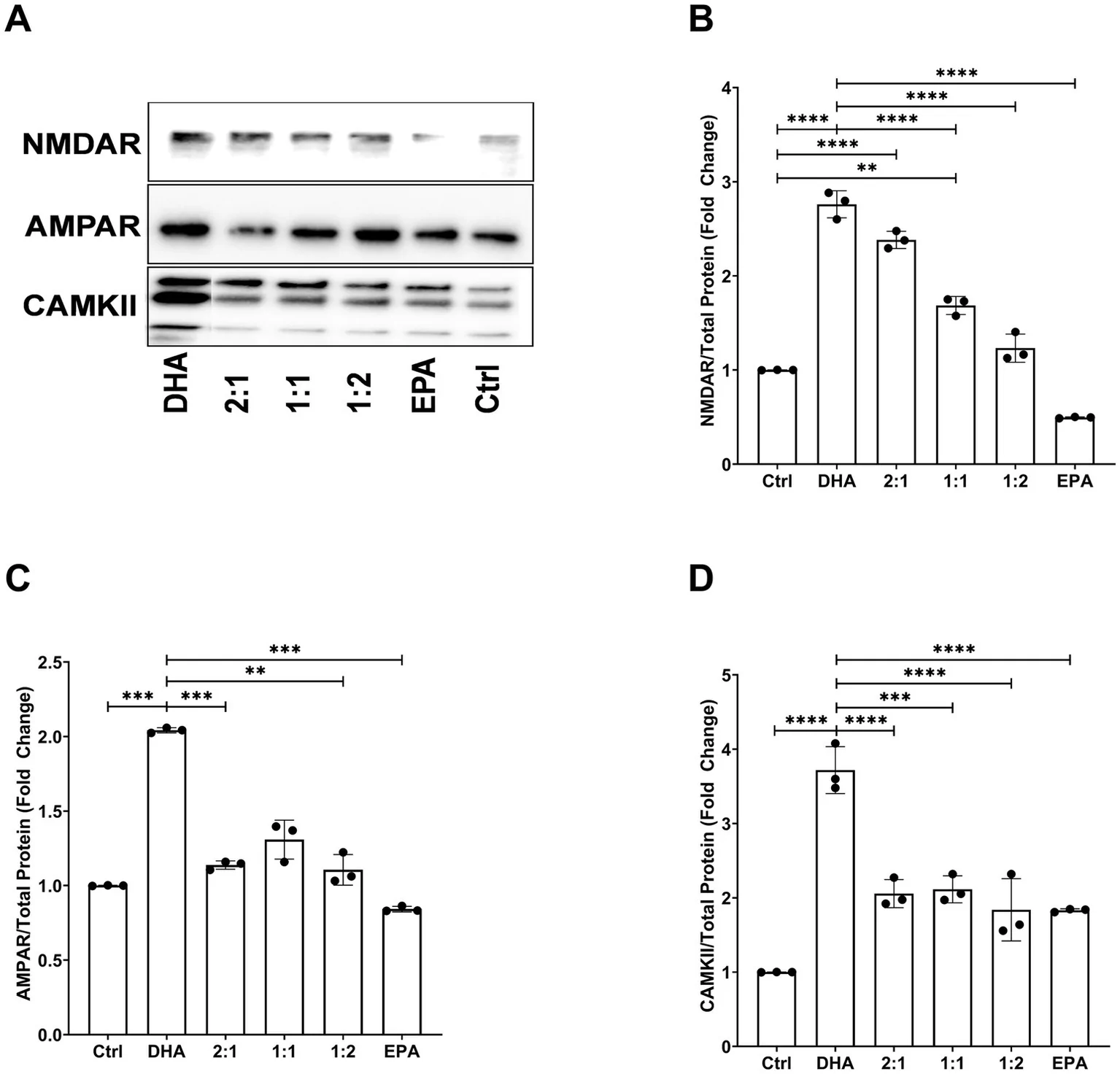
DHA and defined DHA:EPA ratios differentially modulate glutamate receptor and CaMKII expression. **(A)** Representative Western blots of NMDA receptor (NMDAR), AMPA receptor (AMPAR), and CaMKII following 24 h treatment with vehicle control (Ctrl), DHA (50 μM), EPA (50 μM), or DHA:EPA ratios at molar ratios of 2:1, 1:1, and 1:2 (total fatty acid concentration 50 μM). **(B)** NMDAR protein levels normalized to total protein following lipid treatment. DHA and the 2:1 DHA:EPA ratio increased NMDAR expression relative to control and EPA-treated cells, whereas EPA reduced NMDAR levels relative to control. **(C)** AMPAR protein levels normalized to total protein following lipid treatment. DHA increased AMPAR expression relative to control, EPA, and the 2:1 DHA:EPA ratio. **(D)** CaMKII protein levels normalized to total protein following lipid treatment. All lipid-treated groups increased CaMKII expression relative to control, with DHA producing the largest increase. Data are presented as mean ± SD. Individual points represent independent biological replicates (*n* = 3). Statistical analysis was performed using one-way ANOVA followed by Tukey’s multiple comparisons test. ^*^p < 0.05, ^**^p < 0.01, ^***^p < 0.001, ^****^p < 0.0001. Only significant pairwise comparisons are shown.

AMPAR expression followed a similar but less pronounced pattern (Figure 3C). DHA produced the largest increase relative to control (adjusted *p* = 0.0008), with the 1:1 ratio showing a similar numerical response. In contrast, EPA treatment did not significantly alter AMPAR expression relative to control, while the remaining DHA:EPA ratios produced minimal or no detectable change. CaMKII levels increased significantly relative to control following DHA (adjusted *p* < 0.0001), 2:1 DHA:EPA (*p* = 0.0072), 1:1 DHA:EPA (*p* = 0.0028), 1:2 DHA:EPA (*p* = 0.0227), and EPA (*p* = 0.0240) (Figure 3D). DHA produced the strongest response, while EPA- and DHA:EPA-treated conditions showed smaller increases.

Taken together, these findings indicate that glutamatergic receptor and signaling protein responses vary according to DHA:EPA ratio, with DHA producing the strongest overall molecular response. The direction and relative magnitude of changes across all measured endpoints are summarized in Table 1.

**Table 1:** Summary of changes in measured outcomes following treatment with DHA, EPA, and defined DHA:EPA ratios.

Marker/readout	DHA	2:1	1:1	1:2	EPA
MTT metabolic activity	↔	↔	↔	↔	↔
PSD-95	↑↑	↑↑	↑	↑	↔
PSD-95 Puncta Density	↑↑	↑	↑	↑	↔
Total PSD-95 apposed to SYN	↑↑	↑	↑	↑	↓
% PSD-95 apposed to SYN	↑	↔	↔	↔	↓
NMDAR	↑↑↑	↑↑	↑	↑	↔
AMPAR	↑↑	↔	↔	↔	↔
CAMKII	↑↑↑	↑	↑	↑	↑

Arrows indicate the direction and approximate magnitude of the mean change relative to vehicle control and are intended as a qualitative summary rather than indicators of statistical significance. Statistical comparisons are reported in Figures 1–3, Supplementary Figure 2. For MTT-derived metabolic activity, no significant differences were detected among treatment groups. ↑ = modest increase; ↑↑ = strong increase; ↑↑↑ = very strong increase; ↓ = decrease; ↔ = minimal change. DHA, docosahexaenoic acid; EPA, eicosapentaenoic acid; PSD-95, postsynaptic density protein 95; SYN, synaptophysin; NMDAR, N-methyl-D-aspartate receptor; AMPAR, α-amino-3-hydroxy-5-methyl-4-isoxazolepropionic acid receptor; CaMKII, calcium/calmodulin-dependent protein kinase II.

## 4 Discussion

Over the past two decades, long-chain ω-3 fatty acids have been increasingly incorporated into dietary recommendations and studied for cardiovascular and brain health (Calder et al., 2026). The main ω-3 components are the long-chain polyunsaturated fatty acids DHA and EPA, which are present in varying ratios. Although they are structurally similar (DHA has 22 carbons and 6 double bonds, whereas EPA has 20 carbons and 5 double bonds), the two exert distinct effects *in vivo*. EPA, which has a shorter half-life than DHA, is thought to function primarily as a signaling molecule with systemic anti-inflammatory effects (Bazinet and Layé, 2014; Dyall, 2015). In contrast, the more stable DHA pool serves both structural and signaling roles, particularly in the central nervous system, where it supports synaptic health (Aryal et al., 2019; Dyall, 2015). With respect to brain health, it remains unclear whether EPA enhances DHA-associated synaptic effects, attenuates them or is neutral in its impact when present in mixed ω-3 formulations. In this regard, recent work has pointed to a deleterious impact of EPA on the brain’s recovery from neurotrauma (Karakaya et al., 2026).

Notably, ω-3 formulations across experimental and clinical brain-related studies vary substantially in DHA:EPA ratio (Bazinet and Layé, 2014; Brenna and Carlson, 2014; Dyall, 2015) and observed outcomes. For instance, EPA-predominant formulations have been associated with greater efficacy in depression (Sublette et al., 2011), whereas DHA-containing formulations are more commonly used in cognitive and neurodegenerative studies (Freund-Levi et al., 2006; Luchtman and Song, 2013; Yurko-Mauro et al., 2010). These differences suggest that DHA:EPA ratio may influence postsynaptic protein regulation and synaptic organization. Increasing EPA may alter the membrane environment and limit the ability of DHA to promote PSD-95 stabilization (Chen et al., 2013; Kaur et al., 2013; Sherratt and Mason, 2018; Sherratt et al., 2021).

Given our research focus on PSD-95, a key arbiter of synaptic health, we therefore elected to examine the effects of DHA, EPA, and DHA:EPA combinations on PSD-95-related synaptic markers in differentiated human neuronal cells, quantifying PSD-95 abundance, synaptic puncta density, PSD-95 colocalization with SYN, and expression of NMDAR, AMPAR, and CaMKII. DHA treatment in isolation conferred the greatest increases in all these parameters. Across the measured endpoints, DHA produced the strongest overall molecular response, whereas EPA and mixed DHA:EPA conditions produced distinct, generally attenuated profiles. These findings indicate that altering the DHA:EPA ratio changes the pattern of PSD-95-associated and glutamatergic protein responses. These effects occurred without a detectable reduction in MTT-derived metabolic activity. Thus, modifying the DHA:EPA ratio yields distinct postsynaptic outcomes.

### 4.1 Differential regulation of PSD-95 by DHA and EPA

The greater impact of DHA on PSD-95-related outcomes compared with EPA fits with established differences in DHA and EPA membrane incorporation and turnover within the CNS. DHA is among the most abundant polyunsaturated fatty acids in gray matter and can comprise 10–20 percent of the neuronal membrane phospholipids, exceeding 30% in some synaptic or retinal membranes (Brenna and Diau, 2007; Dyall, 2015; Kim et al., 2022).

EPA, in contrast, is usually less than 0.5% of brain fatty acids and is sometimes nearly undetectable in neuronal phospholipids, while EPA accumulates less due to faster turnover (Brenna and Diau, 2007; Chen et al., 2009; Chen et al., 2013; Dyall, 2015; Kaur et al., 2013). Developmental studies have shown increased incorporation of DHA-containing phospholipids in postsynaptic membranes during synaptic maturation, alongside increases in PSD-95 expression and scaffold organization (Cao et al., 2009; Petralia et al., 2005; Swulius et al., 2010; Tulodziecka et al., 2016).

Together, these observations are consistent with the stronger effect of DHA on PSD-95 abundance observed in the present study. The differential responses to DHA and EPA may also reflect differences in intracellular fatty acid handling. Exogenous long-chain PUFAs require activation to acyl-CoA intermediates before incorporation into complex lipids. In neurons, long-chain acyl-CoA synthetase 6 (ACSL6) is an important determinant of DHA enrichment, with ACSL6 deficiency producing substantial reductions in DHA-containing brain phospholipids (Fernandez et al., 2021). Subsequent phospholipid remodeling can occur through deacylation–reacylation pathways, including the Lands cycle; lysophosphatidylethanolamine acyltransferase 2 (LPEAT2) has been shown to incorporate DHA into phospholipids and regulate phospholipid DHA content in neuronal cells (Eto et al., 2020). In addition, phosphatidylserine synthase 2 (PSS2) preferentially utilizes DHA-containing phosphatidylethanolamine substrates, providing a route for enrichment of DHA-containing phosphatidylserine (Kimura and Kim, 2013). These enzymatic processes provide plausible mechanisms by which DHA and EPA could be differentially retained or distributed within neuronal phospholipids; however, these pathways were not directly measured in the present study.

### 4.2 DHA-dominant conditions enhance PSD-95 clustering and SYN colocalization

In addition to increasing total PSD-95 abundance, DHA:EPA ratio altered the extent of synaptic organization of PSD-95. DHA alone increased PSD-95 puncta density and PSD-95 colocalization with SYN, whereas EPA alone failed to increase either measure compared with control. DHA:EPA mixtures produced intermediate responses, with stronger effects under DHA-dominant conditions. This pattern indicates that DHA supports not only PSD-95 expression, but also the clustering and neuritic distribution and SYN colocalization of this scaffold protein. Because total fatty acid concentration was held constant at 50 μM, increasing the EPA proportion necessarily reduced the absolute DHA concentration. The present design therefore cannot distinguish a ratio-specific interaction between DHA and EPA from an effect attributable to reduced DHA exposure. Differential fatty acid incorporation, phospholipid remodeling, and downstream metabolic or signaling responses may additionally contribute. Notably, the reduction in NMDAR abundance observed with EPA alone indicates that EPA was not biologically inert in this system.

PSD-95 localization is central to excitatory synaptic organization because clustered PSD-95 organizes and stabilizes glutamate receptor complexes within the postsynaptic density (Petralia et al., 2005; Swulius et al., 2010). The stronger PSD-95-SYN colocalization observed under DHA-dominant conditions therefore indicates a more organized synaptic profile, not simply a change in total protein abundance. Previous studies show that PSD-95 clustering increases during synaptic maturation and strengthens excitatory contacts (Petralia et al., 2005, Swulius et al., 2010). DHA has also been shown to promote neurite outgrowth, synaptic protein expression, and synaptogenesis in neuronal systems, whereas ω-3 deficiency reduces PSD-95 levels and disrupts synaptic organization (Aryal et al., 2019; Cao et al., 2009; Ikemoto et al., 1997; Madore et al., 2020; Sidhu et al., 2016).

The contrast between DHA- and EPA-containing conditions is noteworthy. EPA alone did not reproduce DHA’s effects on PSD-95 puncta density or PSD-95-SYN colocalization. However, because the total fatty acid concentration was held constant at 50 μM, increasing the proportion of EPA necessarily reduced the absolute DHA concentration. The present design therefore cannot distinguish a ratio-specific interaction between DHA and EPA from an effect attributable to reduced DHA exposure. Differential fatty acid incorporation, phospholipid remodeling, or downstream metabolic and signaling responses may also contribute. Thus, changing the DHA:EPA ratio altered the magnitude of the observed PSD-95 response, but the mechanism underlying this effect remains to be determined.

### 4.3 DHA:EPA ratio shapes glutamatergic receptor and CaMKII expression

DHA-dominant conditions also produced the clearest effects on glutamatergic receptors and signaling proteins. The glutamate receptor and CaMKII data similarly showed the strongest overall response under DHA treatment, although the individual proteins exhibited distinct treatment profiles. The 2:1 DHA:EPA ratio most closely resembled DHA alone, while equimolar and EPA-dominant mixtures produced intermediate responses. Thus, the receptor and CaMKII data reinforce the same hierarchy observed for PSD-95, with DHA producing the strongest postsynaptic response and EPA failing to reproduce this effect.

Although CaMKII is a major regulator of postsynaptic plasticity, it also has established presynaptic functions, including regulation of neurotransmitter release (Pang et al., 2010). Because CaMKII was quantified in whole-cell lysates in the present study, these measurements do not distinguish pre- and postsynaptic CaMKII pools. Accordingly, changes in total CaMKII abundance are interpreted here as a synaptic signaling-related response rather than as a specifically postsynaptic measurement.

Our results are consistent with the coordinated roles of NMDARs, AMPARs, PSD-95, and CaMKII in excitatory synaptic signaling and plasticity. Increased PSD-95 abundance and synaptic localization would be expected to support receptor stabilization and activity-dependent signaling, whereas weaker PSD-95 organization would limit this coordinated postsynaptic response. Prior studies similarly link DHA status to glutamatergic signaling and synaptic plasticity. DHA deficiency reduces postsynaptic scaffold abundance and alters synaptic organization, while dietary DHA supplementation can increase synaptic proteins, including PSD-95, synapsin-1, glutamate receptor subunits, and proteins involved in synaptic plasticity (Aryal et al., 2019; Calon et al., 2004; Cao et al., 2009; Crupi et al., 2013; Madore et al., 2020; Sidhu et al., 2011; Sidhu et al., 2016).

EPA alone did not behave like DHA, failing to strengthen the postsynaptic receptor profile and reducing NMDAR relative to control. This indicates that the postsynaptic response depends on fatty acid identity, not simply ω-3 exposure. This is consistent with the lower incorporation and faster turnover of EPA in brain phospholipids, as well as its functionally distinct signaling roles (Bazinet and Layé, 2014; Chen et al., 2009; Chen et al., 2013; Dyall, 2015). Increasing EPA content was associated with progressively weaker responses under the fixed-total-lipid conditions used here; whether this reflects reduced DHA availability, differential lipid incorporation, or downstream metabolic interactions remains to be determined.

These data indicate that DHA:EPA ratio determines the strength of the glutamatergic postsynaptic response. DHA alone and DHA-dominant formulations produced a coordinated increase in PSD-95, glutamate receptor proteins, and CaMKII, whereas EPA-rich conditions produced weaker or adverse effects on key postsynaptic markers. These findings provide an experimental basis for considering DHA and EPA as non-interchangeable ω-3 species when examining synaptic protein regulation.

More broadly, alterations in PSD-95 expression and postsynaptic scaffold organization have been reported across a range of synaptic disorders, including DLG4-related synaptopathy, autism spectrum disorder, schizophrenia, Alzheimer’s disease, and Fragile X syndrome (Funk et al., 2017; Kindler and Kreienkamp, 2012; Levy et al., 2022; Medina and MacKenzie, 2024; Yuki et al., 2014). In this regard, the coordinated changes in PSD-95, NMDAR, AMPAR, and CaMKII observed under DHA-dominant conditions suggest that membrane lipid composition may influence postsynaptic scaffold stability and glutamatergic signaling in disorders in which these processes are disrupted. Although the present study does not establish therapeutic efficacy, these findings provide a rationale for further investigation of lipid-based approaches in disease-relevant neuronal models.

### 4.4 Study limitations and future directions

Several limitations should be considered when interpreting these findings. First, the experiments used acute 24-h lipid exposure in differentiated SH-SY5Y neurons. The 50 μM treatment used here represents an acute nominal *in vitro* exposure and does not reproduce the delivery, distribution, metabolism, or membrane incorporation of DHA and EPA *in vivo*. Although comparable micromolar DHA concentrations have been used in human neuronal culture studies (Zhao et al., 2019), the possibility that acute lipid loading or cellular adaptation contributes to the observed responses cannot be excluded. In addition, high-micromolar concentrations of unesterified fatty acids may directly influence membrane biophysical properties independent of their subsequent metabolic incorporation (De Santis et al., 2018). This model is useful for comparing DHA:EPA ratio-dependent molecular responses, but it does not capture the cellular diversity, maturation state, or network organization of the human cortex. Evaluation in human iPSC-derived neurons, including genetically defined models of postsynaptic scaffold disorders, will be important to determine whether the DHA-dominant response extends to more mature and disease-relevant neuronal systems.

Because treatment-induced fatty acid incorporation and phospholipid species were not directly measured, the present study cannot determine whether the observed protein responses arise from membrane lipid remodeling, differential fatty acid metabolism, downstream signaling, or a combination of these mechanisms. Lipidomic profiling, membrane-domain analyses, and assessment of PSD-95 interaction partners would help determine how DHA- and EPA-dominant conditions differentially affect the postsynaptic membrane environment.

Third, the study did not directly assess receptor phosphorylation, synaptic transmission, or activity-dependent plasticity. Therefore, the observed increases in PSD-95, NMDAR, AMPAR, and CaMKII should be interpreted as molecular evidence of postsynaptic reorganization rather than direct evidence of enhanced synaptic function. Future studies incorporating electrophysiology, MEA recordings, or activity-dependent stimulation paradigms will be needed to determine whether these molecular changes translate into functional synaptic or network effects.

Finally, the signaling mechanisms responsible for the DHA-dominant response remain to be defined. PI3K/Akt and MAPK/ERK are plausible candidates because both pathways regulate synaptic protein expression and plasticity (Akbar et al., 2005; Kim et al., 2022). Pathway inhibition, time-course analyses, and lipidomic integration would help clarify whether DHA-dominant formulations preferentially engage intracellular signaling programs that support PSD-95 stabilization and glutamatergic receptor organization.

## 5 Conclusion

This study shows that DHA and EPA are not interchangeable regulators of postsynaptic protein organization. DHA produced the strongest increases in PSD-95 abundance, PSD-95 synaptic localization, glutamatergic receptor proteins, and CaMKII, while EPA alone failed to reproduce these effects and reduced NMDAR relative to control. DHA-dominant compositions, particularly the 2:1 DHA:EPA ratio, most closely preserved the DHA response, whereas equimolar and EPA-dominant formulations produced weaker or intermediate effects. These findings indicate that ω-3 effects on synaptic biology depend on fatty acid identity and relative composition rather than total ω-3 exposure alone. In this context, the DHA:EPA ratio represents a modifiable factor for shaping postsynaptic scaffold stability and glutamatergic signaling in disorders characterized by disrupted synaptic organization.

## Data Availability

The raw data supporting the conclusions of this article will be made available by the authors, without undue reservation.

## Supplementary material

Supplementary Figure 1 Stain-Free total-protein membrane images used for Western blot normalization. Full Stain-Free membrane images from the three independent biological replicates used for Western blot analysis: **(A)** biological replicate 1, **(B)** biological replicate 2, and **(C)** biological replicate 3. Each independent experiment contained the complete treatment set: DHA (50 μM), DHA:EPA 2:1, DHA:EPA 1:1, DHA:EPA 1:2, EPA (50 μM), and vehicle control. Target-protein signals were normalized to the total-protein signal from the corresponding Stain-Free membrane lane. Molecular-weight markers are indicated in kDa.

Supplementary Figure 2 Omega-3 fatty acid treatment does not significantly alter MTT-derived metabolic activity in differentiated SH-SY5Y neurons. Differentiated SH-SY5Y neurons were treated for 24 h with vehicle control (Ctrl), DHA (50 μM), EPA (50 μM), or DHA:EPA ratios of 2:1, 1:1, and 1:2 at a total fatty acid concentration of 50 μM. Metabolic activity was assessed using the MTT assay and expressed as fold change relative to vehicle-treated control. Data are presented as mean ± SD from three independent biological replicates (*n* = 3). Group differences were evaluated using one-way ANOVA followed by Tukey’s multiple comparisons test. No significant differences were detected among treatment groups.

Supplementary Figure 3 Neurite-restricted analysis of PSD-95 following DHA and DHA:EPA treatment. **(A)** Representative immunofluorescence images of differentiated SH-SY5Y neurons treated for 24 h with vehicle control (Ctrl), DHA (50 μM), or EPA (50 μM), showing PSD-95 (green), MAP2 (yellow), and merged images with Hoechst-stained nuclei (blue). Scale bar = 20 μm. The analysis overlay shows a representative Columbus-derived MAP2-positive neurite segmentation and PSD-95 puncta detected within the neurite-defined compartment used for quantification. **(B)** PSD-95 fluorescence intensity quantified within MAP2-positive neurites and expressed as fold change relative to vehicle-treated control. **(C)** PSD-95 puncta density within MAP2-positive neurites, normalized to total neurite length and expressed as puncta per 100 μm of neurite. Data are presented as mean ± SD from three independent biological replicates (*n* = 3). Group differences were evaluated using one-way ANOVA followed by Tukey’s multiple comparisons test. Only comparisons with vehicle control are shown for clarity. ^*^*p* < 0.05, ^**^*p* < 0.01, ^***^*p* < 0.001, ^****^*p* < 0.0001.
